# A Review of Recent Advancement in Integrating Omics Data with Literature Mining towards Biomedical Discoveries

**DOI:** 10.1155/2017/6213474

**Published:** 2017-02-26

**Authors:** Kalpana Raja, Matthew Patrick, Yilin Gao, Desmond Madu, Yuyang Yang, Lam C. Tsoi

**Affiliations:** ^1^Department of Dermatology, University of Michigan Medical School, Ann Arbor, MI, USA; ^2^Department of Computational Medicine & Bioinformatics, University of Michigan Medical School, Ann Arbor, MI, USA; ^3^Department of Biostatistics, University of Michigan, Ann Arbor, MI, USA

## Abstract

In the past decade, the volume of “omics” data generated by the different high-throughput technologies has expanded exponentially. The managing, storing, and analyzing of this big data have been a great challenge for the researchers, especially when moving towards the goal of generating testable data-driven hypotheses, which has been the promise of the high-throughput experimental techniques. Different bioinformatics approaches have been developed to streamline the downstream analyzes by providing independent information to interpret and provide biological inference. Text mining (also known as literature mining) is one of the commonly used approaches for automated generation of biological knowledge from the huge number of published articles. In this review paper, we discuss the recent advancement in approaches that integrate results from omics data and information generated from text mining approaches to uncover novel biomedical information.

## 1. Introduction

The advances in biotechnology have allowed biomedical research to answer efficiently important biological questions in the different omics scales: genetics, genomics, transcriptomics, epigenomics, proteomics, and metabolomics [1–4]. The omics data can characterize the behaviors of cells, tissues, and organs at the molecular level and allow the comprehensive understanding for the etiology of human diseases. Among the various omics studies, genetic and genomic studies are widely adopted in biomedical research to discover new genes or susceptibility loci associated with different human traits or diseases [5, 6]. Proteomic study is concerned with the structure, function, and modification of proteins expressed in a biological system, specifically the posttranscriptional modifications such as phosphorylation, methylation, and acetylation, which lead to transcription and translation of the same genome into various types of proteomes [7, 8]. Epigenomic study has attracted great attention in the last 5 years. It characterizes the epigenetic modifications of the genome and aims to understand the regulations of the gene expression. Transcriptomic study, in turn, enables the genome-wide assessment of gene expression patterns in cells and tissues by studying the complete set of RNA transcriptomes [9]. Finally, metabolomic study characterizes the metabolites present in cell, tissue, and body fluid and identifies the fluctuation of these metabolites in various disease conditions [10]. The different types of omics studies accumulate a huge volume of data through high-throughput sequencing experiments and provide insights towards the cellular and metabolic processes related to disease diagnoses, treatment, and prevention.

According to the PubMed, over 36,000 research articles have been published in the past ten years and annotated by at least one of the above “omics” experiments (by using the following search phrase: “(genomics [MeSH] OR proteomics [MeSH] OR metabolomics [MeSH] OR transcriptomics [MeSH]) AND humans [MeSH]”). The interest in omics studies has not declined and their applications are evident from the publications in recent years, when compared to only over 10,000 research articles published prior to 2006 by using the same search phrase. However, the acquired data raises various significant challenges: (i) the interpretation of high-throughput results; (ii) the translation of biological data to clinical application; (iii) the data handling, storage, and sharing issues; and (iv) the reproducibility when comparing between different experiments [11, 12]. Among these, the last challenge has been a long-lasting issue, most likely due to the potential discrepancies in processing and interpreting the high-throughput data or due to “cherry-picking” approach to subjectively focus on the components that are indeed false positives. The traditional strategies to overcome these challenges are to conduct extensive literature search and seek professional opinions from domain experts to decipher the mechanism and then conduct downstream experiments to verify the findings. However, this has proven to be time consuming and subjective and has not been a common practice when researchers publish their results from high-throughput experiments. On the other hand, automated approaches have gained much interest in recent years to annotate gene functions [13], to identify biomarkers [14], and to explore genetic mutations [15]. Text mining (also known as literature mining) is a technique that has been used to retrieve and process research articles from PubMed database and can summarize biomedical information present across articles. In molecular biology, text mining is typically used to retrieve relevant documents, prioritize the documents, extract the biomedical concepts (e.g., genes, proteins, cell, tissue, and cell-type), and extract the causal relationships between concepts [16, 17]. Text mining can significantly decrease the time and effort required, compared with traditional labor-intensive approaches.

In this review, we first discuss the various omics techniques used in healthcare and summarize the recent advances in utilizing text mining approaches to facilitate the interpretation and translation of these omics data. We then focus on biomedical literature mining and clinical text mining and further describe the challenges involved in integrating the knowledge from different resources to enhance the biomedical research. Finally, we explain the recent methods to integrate omics and biomedical literature mining data in order to uncover novel biomedical information.

## 2. The Study of “Omics”

Traditionally, “omics” corresponds to the study of four major biomolecules: genes, proteins, transcriptomes, and metabolites [4]. Since the discovery of DNA [31], much interest has been gained towards understanding the roles of genes and proteins in cellular functions and transduction. Healthcare is considered to vary from one individual to another based on his genome, proteome, transcriptome, and metabolome. The digital revolution has paved the way for integrating patient omics data with the findings in literature for the discovery of novel biomarkers and drug targets [32–34]. Therefore, the study of omics has expanded beyond these four major omics studies, and Table 1 summarizes the various types of omics data applied to biomedical discoveries. The study of omics has introduced the realm of big data to biomedicine [35, 36]. While the first human genome project took more than a decade to complete and involved $3 billion dollars, the entire genome can be sequenced and analyzed within hours for ~$1000 now. Thus, biomedical projects are now possible to generate information at the petabyte (i.e., 1,012 bytes) scale. Nevertheless, the greatest challenge is the large-scale data analysis and its integration with clinical data available in patient electronic health records (EHR) [37].

Cloud [38] and parallel computing [39] are currently used in omics research to handle the huge volume of data. Cloud computing is described as a network of computers connected together through the Internet for effective processing. It is available remotely, through cloud computing providers (e.g., Microsoft, Google, and Amazon), and researchers have an option to make use of it at an affordable cost. Parallel computing speeds up the processing time using the same hardware and Internet setup. The combined approach of using cloud computing and parallel computing together is capable of processing omics data in a feasible time [40, 41]. Other high performance computing platforms include clusters [42], grid computing [43], and graphical processing units [44]. Processing omics data and applying bioinformatics models to the data require expertise to integrate computational, biological, mathematical, and statistical knowledge.

## 3. Text Mining

PubMed database is a main repository for biomedical literature and contains over 26 million articles. The number of articles being published and indexed by PubMed is increasing exponentially, and therefore text mining has become an attractive (and standard) approach in mining literature data when comparing with the traditional labor-intensive strategies. Researchers use the text mining approach to tackle information overload, both in biomedical and in general areas of big data collection, because it automates data retrieval and information extraction from the unstructured biomedical texts to reveal novel information [45, 46]. While information extraction examines the relationships between specific kinds of information contained within or between documents, information retrieval focuses on summarizing data from the larger units of documents [47]. Another automated approach to deal with unstructured data is Natural Language Processing (NLP). While text mining concentrates on solving a specific problem in a particular domain, NLP attempts to understand the text as a whole [48]. Recently, text mining and NLP have been used to address different biological questions in omics research [49].

### 3.1. Biomedical Literature Mining

The era of applying text mining approaches to biology and biomedical fields came into existence in 1999. It was first applied to the biomedical domain for gene expression profiling [50], as well as the extraction and visualization of protein-protein interaction [51]. It emerged as a hybrid discipline from the edges of three major fields, namely, bioinformatics, information science, and computational linguistics. Biomedical literature mining is concerned with the identification and extraction of biomedical concepts (e.g., genes, proteins, DNA/RNA, cells, and cell types) and their functional relationships [17]. The major tasks include (i) document retrieval and prioritization (gathering and prioritizing the relevant documents); (ii) information extraction (extracting information of interest from the retrieved document); (iii) knowledge discovery (discovering new biological event or relationship among the biomedical concepts); and (iv) knowledge summarization (summarizing the knowledge available across the documents). A brief description of the biomedical literature mining tasks is listed as follows. 

*Biomedical Text Mining Tasks*

*Document Retrieval*. The process of extracting relevant documents from a large collection is called document retrieval or information retrieval [52]. The two basic strategies applied are query-based and document-based retrieval. In query-based retrieval, documents matching with the user specified query are retrieved. In document-based retrieval, a ranked list of documents similar to a document of interest is retrieved. 

*Document Prioritization*. The retrieved documents are usually prioritized to get the most relevant document. Many biomedical document retrieval systems achieve prioritization based on certain parameters including journal-related metrics (e.g., impact factor, citation count) [53] and MeSH index [54, 55] for biomedical articles. The similarity between the documents is estimated with various similarity measurements (e.g., Jaccard similarity, cosine similarity) [56]. 

*Information Extraction*. This task aims to extract and present the information in a structured format. Concept extraction and relation/event extraction are the two major components of information extraction [57, 58]. While concept extraction automatically identifies the biomedical concepts present in the articles, relation/event extraction is used to predict the relationship or biological event (e.g., phosphorylation) between the concepts [59, 60]. 

*Knowledge Discovery*. It is a nontrivial process to discover novel and potentially useful biological information from the structured text obtained from information extraction. Knowledge discovery uses techniques from a wide range of disciplines such as artificial intelligence, machine learning, pattern recognition, data mining, and statistics [61]. Both information extraction and knowledge discovery find their application in database curation [62, 63] and pathway construction [64, 65]. 

*Knowledge Summarization*. The purpose of knowledge summarization is to generate information for a given topic from one or multiple documents. The approach aims to reduce the source text to express the most important key points through content reduction selection and/or generalization [66]. Although knowledge summarization helps to manage the information overload, the state of the art is still open to research to develop more sophisticated approaches that increase the likelihood of identifying the information. 

*Hypothesis Generation*. An important task of text mining is hypothesis generation to predict unknown biomedical facts from biomedical articles. These hypotheses are useful in designing experiments or explaining existing experimental results [67].

Conventional text mining approaches process PubMed abstracts rather than the full-text articles and fail to mine the information not in abstracts. Recently, text mining from the full-text articles is gaining more interest [59]. However, it involves many challenges: (1) the availability of full-text articles is limited (4 million full-text articles in PubMed Central versus 26 million abstracts in PubMed); (2) text mining within tables, figures, and equations is complicated; and (3) information redundancy within the articles. An automated text mining system is generally evaluated using a standard corpus (Table 2). However, the availability of standard corpora in biomedical domain is limited because its generation is expensive, time consuming, and requires domain experts. In general, a gold standard is developed within the research groups when the standard corpora are not available, but mostly not available to other researchers. The text mining systems are commonly evaluated using precision, recall, and f-score. Precision is defined as the relevance accuracy, recall is defined as the retrieval accuracy, and f-score is defined as the harmonic mean of precision and recall [56].

### 3.2. Clinical Text Mining

Electronic health records, discharge summaries, and clinical narratives of patients are rich in information that could be useful for improving the healthcare. In addition, the information is also available from the transcription of dictations, direct entry by clinicians/physicians, or speech recognition software. The encoding of structural information from the clinical resources is useful to clinicians and researchers. For example, automated high-throughput clinical applications can be developed to support clinicians' information needs [68]. However, manual encoding is expensive and limited to primary and secondary diagnoses. Clinical text mining, also known as clinical NLP or Medical Language Processing (or simply MLP), is suggested as a potential technology by Institute of Medicine for mining clinical resources. The tasks described above in biomedical literature mining are applicable to clinical text mining and include additional subtasks [69]: (i) negation recognition (e.g., “patient denies on developing rashes”), (ii) temporal extraction (e.g., “small bumps noticed last year”), and (iii) patient-event relationship (e.g., “patient mother had arthritis”).

The modern healthcare relies on big data analytics for integrating, organizing, and utilizing different pharmacological or clinical information. A hybrid approach to combine patient genomic data and electronic health record information is expanding as the future vision of healthcare. The omics data has become an emerging tool for diagnosis/clinical investigations of common and rare diseases and helps in clinical decision making (i.e., selecting the best possible treatments for patients). Genome-Wide Association Study (GWAS), also known as Whole Genome Association Study (WGAS), is a relatively new approach for identifying genes (i.e., loci associated with human traits) through rapid scanning of markers across whole DNA or genome [70]. GWAS has been applied also to cancer research for drug repositioning [71], prioritizing susceptible genes in Crohn's disease [72], and analyzing the human variants in the area of precision medicine [73]. As an example, the Michigan Genomics Initiatives (MGI) at the University of Michigan has developed an institutional based DNA and genetics repository combined with patient phenotype. The project aims to bring awareness to each patient/participant about the disease development and response to treatments for better health and wellness. The current studies at MGI include analgesics outcome study (AOS), understanding opioid use in chronic pain patients, a pivotal study on high-frequency nerve block for postamputation pain, Michigan body map (MBM), and positive piggy bag (https://www.michigangenomics.org/).

Clinical text mining faces the following specific challenges: (1) access to patient EHR requires permission from Institutional Review Board (IRB); (2) personal details of the patients should be deidentified; (3) mining approaches depend on the types of clinical documents (e.g., EHR, discharge summary, medical billing, and clinical narratives); (4) mining of dosage information, different types of formulations, and temporal information is demanded; and (5) spelling mistakes and grammatical errors are common in clinical text [69]. The state of the art for both biomedical literature mining and clinical text mining is still open with many challenges and requires more sophisticated and robust approaches.

## 4. Role of Text Mining in Omics Study

Relationship between concepts of the same kind (e.g., gene-gene) or different kind (e.g., gene-disease) is commonly known as “event” [74]. The events are useful to identify many clinical facts such as disease onset and response to drug treatment. Overwhelming of biomedical articles from omics research has accumulated abundance of information and requires advanced event extraction systems to support the complexity of available information and coverage of varieties of biomedical subdomains [16]. Text mining approaches do not replace the manual curation of biomedical information but support speeding up the process by several-fold [75, 76]. In this section we describe the various text mining approaches developed for mining omics related information.

### 4.1. Genomics and Text Mining

In the current era of genomics, text mining plays an important role in mining gene-gene interactions [77, 78] and other gene involved interactions (e.g., gene-chemical, gene-disease) [79, 80] to support integrative analysis of gene expression [81, 82], pathway construction [83, 84], ontology development [85], and database annotation [62, 86, 87].

Genes encode proteins and proteins enroll in various biological functions by interacting with other proteins. This encoding process is defined in two steps: transcription (i.e., DNA to RNA) and translation (RNA to protein). Many cellular processes are regulated by microRNA through mRNA degradation and suppression of gene expression such that the protein synthesis is interrupted. This is the fundamental of genomics. In genomics, gene function is assessed from the involvement of genes/proteins in biochemical pathways. The functional genomics is a revolutionary area in text mining where the gene/protein mentions in the biomedical articles and their relationship are considered to be important. Furthermore, gene and protein names are highly complex and text mining has contributed to their recognition in the unstructured text [57, 58].

Different text mining implementations for exploring the finding of genome research have been developed in the past decade. miRTex is a text mining system developed for mining experimentally validated microRNA gene targets from PubMed articles. The system has been successfully implemented to identify the Triple Negative Breast Cancer related genes that are regulated by microRNAs [81]. More sophisticated approaches integrate gene expressions from microarray experiments, biomedical data extracted by text mining, and gene interaction data to predict gene-based drug indications [82]. A similar approach [87] attempts to support manual curation of links between biological databases such as Gene Expression Omnibus (GEO) and PubMed database. Another approach [88] combines text mining data with microarray data for discovering disease-gene association by using unsupervised clustering. The gene-drug interaction information extracted by text mining is used to predict the drug-drug interaction [89]. Above all, the researchers have attempted to use text mining for annotating genome function with gene ontology [90]. Thus, text mining and genomics together uncover much biomedical information that was previously unknown.

### 4.2. Proteomics and Text Mining

Protein-protein interaction is important to explore the mechanism involved in biological processes and onset of diseases [91]. Intact [92], BIND [93], MIND [94], and DIP [95] are the major databases available for protein-protein interaction. These databases are manually curated by the domain experts, but a larger portion of information is still available only in the biomedical literature. Text mining provides a bridge to cover the gap existing between the manual curation and information hidden in the literature. The approaches to extract protein-protein interaction range from simple rule-based systems and cooccurrence systems to more sophisticated NLP methods [60] and machine learning systems [96]. Apart from protein-protein interaction extraction systems, text mining also provides automated approaches for extracting posttranslational modification of proteins such as protein phosphorylation [59].

### 4.3. Transcriptomics, Metabolomics, and Text Mining

Text mining approaches for transcriptomics and metabolomics are limited. One major fact is that these two areas of genomics are comparatively new when compared to genomics and proteomics. A recent study compares the metagenome characteristics of healthy individuals with autism patients to analyze the enzymes involved [97]. The computational approach uses text mining for genomics and metabolomics information extraction. A web-based tool called 3Omics is available for integrating, comparing, analyzing, and visualizing data from transcriptomics, metabolomics, and proteomics [98]. Another tool called Babelomics integrates transcriptomics, proteomics, and genomics data to uncover the underlying function profiles [99]. Thus, a wide variety of hidden biomedical information within the omics data are extracted and predicted through text mining.

## 5. Conclusion

In this review, we summarized the current state of the art in omics research and contribution of text mining approaches to uncover the omics related biomedical information hidden within the published articles. We discussed the core concepts of omics and the challenges involved in storing and analyzing the huge volume of omics data generated from high-throughput experiments. We also highlighted the use of computer techniques such as parallel processing and cloud computing to manage omics data and elaborated on text mining approaches for biomedical literature and clinical text with emphasis on omics. While the omics approach is emerging to be commonly used practice for basic science or clinical diagnosis technique, it is imminent to note that data interpretation and translation is the bottleneck. The advances in text mining can be useful to resolve the challenges with the omics data and further support in novel biomedical discoveries.

## Figures and Tables

**Table 1:** Omics and biomedical applications.

	Omics	Study topic	Biomedical applications^†^
Genetics/molecular genetics	Genomics	Genes	Gencode, Entrez Gene
	Epigenomics	Epigenetics modifications	Gene Express Omnibus
	Exposomics	Disease-causing environmental factors	Comparative Toxicogenomics Database
	Exomics	Exons in a genome	ICE—a human splice sites database
	ORFeomics	Open Reading Frame (ORF)	—
	Phenomics	Phenotypes	Human Phenotype Ontology
	Pharmacogenomics	Impact of genes on individual's response to drugs	PharmGKB
	Pharmacogenetics	SNPs and their impact on pharmacodynamics and pharmacokinetics	PharmGKB
	Toxicogenomics	Genes response to toxic substances	Comparative Toxicogenomics Database

Molecular biology	Proteomics	Proteins and amino acids	Proteomics Identifications Database (PRIDE)
	Metabolomics	Metabolites	HMDB: Human Metabolome Database
	Transcriptomics	Transcripts (i.e., rRNA, mRNA, tRNA, and microRNA)	Human Transcriptome Map
	Ionomics	Inorganic biomolecules	—
	Kinomics	Protein kinases	KinBase database and KinWeb database
	Metagenomics	Genetic material from multiple organisms	MG-RAST
	Regulomics	Transcription factors and other biomolecules involved in the regulation of gene expression	miRegulome
	Toponomics	Cell and tissue structure	—

Medicine	Trialomics	Human interventional trials from clinical trials	—
	Connectomics	Structural and functional connectivity in brain	—
	Interactomics	Interferons	CREDO

^†^The list shows example applications.

**Table 2:** Standard corpora for omics domain.

Corpus	Text mining evaluation task	Brief introduction
JNLPBA (Joint Workshop on NLP in Biomedicine and Its Applications) [18]	Gene/protein concept extraction	The corpus consists of 2,000 PubMed abstracts as training data and 404 PubMed abstracts as test data.

BioCreAtivE 2004 Task 1A dataset [19]	Gene/protein concept extraction	The corpus consists of 15,000 PubMed sentences as training data and 5,000 PubMed sentences as test data.

BioCreAtivE 2 Gene Mention (GM) dataset [20]	Gene/protein concept extraction	The corpus consists of 15,000 PubMed sentences as training data and 5,000 PubMed sentences as test data.

AIMED [21]	Protein-protein interaction	The corpus consists of 225 PubMed abstracts that contain 1,987 sentences with 4,075 protein mentions.

HPRD50 (Human Protein Reference Database) [22]	Protein-protein interaction	The corpus consists of sentences with protein-protein interaction from 50 PubMed abstracts.

BioInfer (Bio Information Extraction Resource) [23]	Protein, gene, and RNA relationships	The corpus consists of 1100 sentences annotated with concept names, relationships, and syntactic dependencies.

IEPA (Interaction Extraction Performance Assessment) [24]	Protein-protein interaction	The corpus consists of more than 200 PubMed sentences annotated with protein-protein interaction.

BioCreAtivE 2.5 Elsevier Corpus [25]	Protein-protein interaction	The corpus consists of 61 PubMed articles as training data and 62 PubMed articles as test data.

BC4GO Corpus [26]	Gene ontology	The corpus consists of 1356 distinct GO terms from 200 PubMed articles.

GREC Corpus [27]	Gene regulation and gene expression events	The corpus consists of 240 PubMed abstracts with annotations on gene regulation and gene expression events.

GETM [28]	Gene expression events	The corpus consists of 150 PubMed abstracts with annotation for gene expression events.

AnEM [29]	Tissue, cell, developing anatomical structure, cellular component	The corpus consists of 500 PubMed sentences with annotations on variety of biomedical concepts.

CellFinder Corpus [30]	Anatomical parts, cell lines, cell types, species, and cell components	The corpus consists of annotations from 10 full-text PubMed articles.
